# Lentivirus-mediated CDglyTK gene-modified free flaps by intra-artery perfusion show targeted therapeutic efficacy in rat model of breast cancer

**DOI:** 10.1186/s12885-019-6111-5

**Published:** 2019-09-14

**Authors:** Jianhua Zhang, Yuanbo Liu, Mengqing Zang, Shan Zhu, Bo Chen, Shanshan Li, Bingjian Xue, Li Yan

**Affiliations:** 1 Department of Plastic and Reconstructive Surgery, Plastic Surgery Hospital, Chinese Academy of Medical Sciences and Peking Union Medical College, Ba-Da-Chu Road 33#, Beijing, 100144 People’s Republic of China; 2 Research Center of Plastic Surgery Hospital, Chinese Academy of Medical Sciences and Peking Union Medical College, Ba-Da-Chu Road 33#, Beijing, 100144 People’s Republic of China

**Keywords:** Suicide gene therapy, Lentiviral vector, CDglyTK gene, Free flap, Intra-artery perfusion, Breast cancer

## Abstract

**Background:**

Free flap-mediated gene therapy in the tumor bed following surgical resection is a promising approach in cancer targeted treatment of residual disease. We investigated the selective killing efficacy of a lentivirus-mediated cytosine deaminase-thymidine kinase (CDglyTK) gene in transplanted breast cancer delivered into a free flap by intra-artery perfusion.

**Methods:**

Proliferation, apoptosis, and cell cycle of rat SHZ-88 breast cancer cells transfected with a lentivirus-mediated CD/TK gene were measured following treatment with ganciclovir and 5-flucytosine in vitro. A model of residual disease of breast cancer in a rat superficial inferior epigastric artery (SIEA) flap model was used to study the therapeutic potential of a double suicide CD/TK and prodrug system in vivo.

**Results:**

Killing efficacy of the double suicide CD/TK and prodrug system on SHZ-88 cells was mediated by increased apoptosis and cell cycle arrest at the G1 phase with significant bystander effect. Following recombinant lentivirus transfection of rat SIEA flap by intra-artery perfusion, CD/TK gene expression was limited to the flap, and the volume and weight of transplanted tumors were significantly reduced without observable toxicity.

**Conclusions:**

SIEA flaps transfected with a lentivirus-mediated CDglyTK gene by intra-artery perfusion effectively suppress transplanted breast tumor growth without obvious systemic toxic effects in rats.

**Electronic supplementary material:**

The online version of this article (10.1186/s12885-019-6111-5) contains supplementary material, which is available to authorized users.

## Background

Breast cancer is the most frequent malignant disease in women worldwide, with nearly 1.4 million new cases annually and an estimated 460,000 deaths per year [1, 2]. Although radical resection is the mainstay of treatment for breast cancer, there is a high risk of locoregional tumor recurrence after ablative surgery [3]. In addition, when such excision takes place, it can result in extensive soft tissue defects and loss of important aesthetic form that often needs microvascular free-tissue transfer for reconstruction, which has gained worldwide acceptance as the primary method for reconstructing post-oncological defects with low overall morbidities [4, 5]. However, free flaps simply reconstruct surgical defects and do not provide direct therapeutic benefits against the underlying cancer or withstand the toxic effects of adjuvant treatments, such as chemotherapy or radiotherapy, after surgical operation [6].

At present, strategies for free flap-mediated gene therapy include suicide gene therapy, immunogene therapy, genetic radionuclide therapy, and free-flap radioprotection [6]. Among them, suicide gene therapy, also known as virus-directed enzyme prodrug therapy (VDEPT), with its particular mechanism, has been increasingly addressed in research. Following transfection of a suicide gene from bacteria or viruses into tumor cells via genetic engineering, this therapeutic gene could encode a specific enzyme in infected cells, which metabolizes an available nontoxic prodrug into an active cytotoxic agent that kills target cells [7, 8] . In addition, VDEPT is known for its intense bystander cytotoxicity effect, in which significant toxicity from the converted prodrug can diffuse to neighboring non-transfected cells [9]. There are a variety of suicide genes, of which the *Escherichia coli* bacterial cytosine deaminase (CD) gene and the herpes simplex virus (HSV)-thymidine kinase (TK) gene have been widely studied. CD metabolizes 5-flucytosine (5-FC) into 5-fluorouracile (5-FU), thereby inhibiting synthesis of DNA and RNA [10]. TK converts ganciclovir (GCV) into the cytotoxic ganciclovir-triphosphate, which inhibits DNA polymerase [11] . Importantly, it has been demonstrated that the cell-killing effect of the combined use of a CD/5-FC system and a HSV-TK/GCV system is much stronger in target cells compared to that found using either system alone [12]. VDEPT may be particularly suited for delivery through a free flap because the production of cytotoxic metabolites will be distributed directly over the tumor bed.

Proliferation of MCF7 human breast cancer cells transfected with an adenovirus-mediated CDglyTK double suicide gene was significantly suppressed by pre-treatment with the prodrugs 5-FC and GCV [12]. Further, adenovirally delivered enzyme prodrug therapy with a TK suicide gene in the superficial inferior epigastric artery (SIEA) flap shows therapeutic efficacy in rat models of glioma [13]. However, there has been no report regarding the treatment effect of a lentivirus-mediated CDglyTK double suicide gene in the SIEP of a rat breast cancer model. In this study, we examined the anti-tumor effect and systemic toxicity of a CD/TK double suicide fusion gene in microvascular free flaps transfected by intra-perfusion on a rat model of breast cancer.

## Methods

### Cell line and animals

The rat breast cancer cell line (SHZ-88) was provided by the Cell Resource Center, Shanghai Institutes for Biological Sciences at the Chinese Academy of Sciences. Cells were cultured in RPMI-1640 Medium Modified (without calcium nitrate) supplemented with 10% fetal bovine serum (Gibco by Life Technologies, Grand Island,NY, USA), 100 U/mL penicillin, and 100 μg/mL streptomycin at 37 °C in 5% CO_2_.

Adult female Sprague-Dawley rats (Vital River, Beijing, China) weighing 250 to 350 g were used for this study, and were purchased from the animal center of Academy of Military Medical Sciences (Beijing, China). The rats were housed under specific pathogen-free conditions. All animal experiments were approved by the Institutional Animal Care and Use Committee of the Plastic Surgery Hospital, and our study were approved by the Ethics Committee of the Plastic Surgery Hospital at the Chinese Academy of Medical Sciences & Peking Union Medical College (Reference number: CZ2015004). The rats were sacrificed by carbon dioxide anesthesia after the procedures.

### Lentivector packing and titration

Lentiviral vectors (lentivectors) were produced by standard transient transfection of a three-plasmid system into packaging cells (HEK293 cell line was obtained from the Institute of Basic Medical Sciences, Chinese Academy of Medical Sciences.). The expression plasmid, control plasmid, and two packaging plasmids were pLVX-CD/TK-ZsGreen, pLVX-IRES-ZsGreen, psPAX2, and pMD2.G, respectively. Recombinant lentivirus was harvested by collecting the supernatant of a virus-producing cell culture and concentrated by ultracentrifugation. The virus titer was determined by: virus titer = the number of green fluorescence protein (GFP)-positive cells / the infective dose of the recombinant virus with 10-fold dilution.

### Transfection of SHZ-88 cells with lentivector co-expressing CD and TK genes

SHZ-88 cells (5 × 10^5^) were seeded and infected with recombinant lentivirus (LV-CMV-CDglyTK or LV-CMV-GFP) at multiplicities of infection (MOI) of 20, 50, 100, and 200. Following a 48-h incubation, the GFP-positive cell percentage was counted using fluorescence microscopy.

### Effect of recombinant lentivirus on SHZ-88 cell growth

SHZ-88 cells (5 × 10^5^) were separately transfected with LV-CMV-CDglyTK or LV-CMV-GFP at MOIs of 0, 50, 100, or 200 for 48 h. Afterwards, infected cells (3 × 10^3^ cells/well) were seeded in 96-well plates. Cell viability was measured using the Cell Counting Kit-8 (CCK-8) solution (Dojindo, Japan) according to the manufacturer’s instructions.

### Reverse transcriptase polymerase chain reaction analysis

SHZ-88 cells (5 × 10^5^) were infected with LV-CMV-CDglyTK or LV-CMV-GFP at an MOI of 100 for 48 h. RNA was then extracted using TRIzol (Life Technologies Corporation, Carlsbad, CA, USA) and reverse transcriptase polymerase chain reaction (RT-PCR) was performed according to the manufacturer’s protocol using the following forward and reverse primers: CD/TK gene (forward, 5′-TGCTTCAGCCGCTACCC-3′; reverse, 5′-AGTTCACCTTGATGCCGTTC-3′) and β-actin (forward, 5′-AGCCATCCAGGCTGTGTTGT-3′; reverse, 5′-CAGCTGTGGTGGTGAAGCTG-3′). The amplification cycles were: 95 °C for 5 min, followed by 30 cycles at 95 °C for 30 s, 56 °C for 30 s, and 72 °C for 30 s, and a final extension at 72 °C for 5 min. Amplified PCR products were electrophoresed and imaged under ultraviolet light.

### Western blot analysis

Total protein was extracted from SHZ-88 cells transfected with recombinant lentivirus using a protein extraction reagent (Sigma-Aldrich, St Louis, MO, USA) according to manufacturer’s instructions. Sample total proteins (15 μg/well) were electrophoresed by 10% sodium dodecyl sulfate polyacrylamide gel electrophoresis and transferred to polyvinylidene fluoride membranes at 200 mA for 2 h. CD polyclonal antibody (GeneTex Biotechnology, Irvine, CA, USA) or TK polyclonal antibody (Santa Cruz Biotechnology, Santa Cruz, CA, USA) were applied at 1:1000 dilutions, and a goat anti-rabbit or goat anti-mouse horseradish peroxidase (1,10,000, Jackson ImmunoResearch, West Grove, PA, USA) was used as the secondary antibody. Blots were detected using an ECL detection kit and intensities of the protein bands were normalized to β-actin using a Gel Image system ver.4.00 (Tanon, Shanghai, China).

### Cell viability assay

SHZ-88 cells were infected with LV-CMV-CD/TK or LV-CMV-GFP at an MOI of 100 for 48 h and treated with 5-FC and GCV (Sigma-Aldrich) separately or in combination. Cell viability was measured using CCK-8 solution (Dojindo) according to the manufacturer’s protocol and as described previously.

### Apoptosis and cell-cycle analysis

SHZ-88 cells were randomly divided into three groups: a LV-CDglyTK gene-infected group (LV-CDglyTK group), an empty lentivirus-infected group (LV-GFP group), and a control group. Cells of the LV-CDglyTK and LV-GFP groups were respectively transduced with LV-CMV-CDglyTK and LV-CMV-GFP at an MOI of 100 for 48 h. Cells were then treated with 5-FC + GCV (600 mg/L and 60 mg/L, respectively) for 48 h. Apoptosis was measured using the Muse Annexin V and Dead Cell Assay Kit (Merck Millipore, Burlington, MA, USA) according to manufacturer’s instructions, and cell cycle analysis was performed using the Muse Annexin V and Dead Cell Assay Kit (Merck Millipore) and Muse Cell Analyzer according to manufacturer’s instructions.

### Bystander kill assay

SHZ-88cells (5 × 10^5^) were infected with LV-CMV-CDglyTK or LV-CMV-GFP at an MOI of 100 and treated with 5-FC + GCV (600 mg/L and 60 mg/L, respectively) for 48 h. Next, non-transfected SHZ-88 cells were seeded in wells containing supernatant from infected cells, and cell viability was measured 48-h later by CCK-8 assay.

### LV-CDglyTK transfection of SIEA flaps

**Fig. 1:**
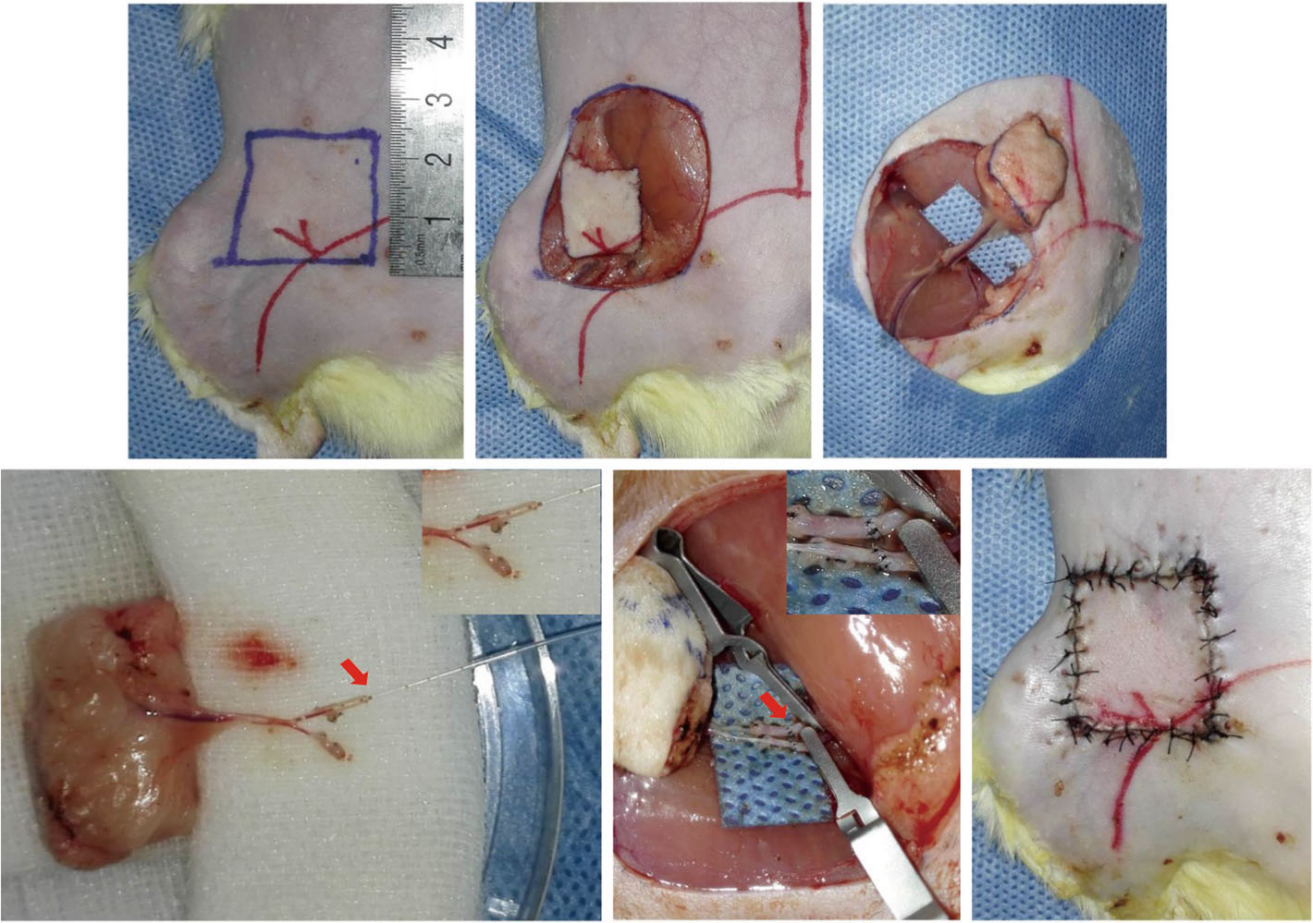
Construction of superficial inferior epigastric (SIEA) flaps transfected with LV-CMV-CDglyTK in a Sprague-Dawley rat model. (upper left panel) The SIEA flap was designed with a 2.5 × 2.5-cm skin paddle. (upper middle panel and upper right panel) The SIEA flap was dissected carefully as described previously. (lower left panel) After the flap pedicle was divided, the afferent artery to the flap was catheterized with a 0.4 mm micro cannula. (lower middle panel) After perfusion of recombinant virus or phosphate-buffered saline through the afferent artery and incubated, the native vessels of the flap were re-anastomosed. (lower right panel) The flap was oriented in its original position in the groin and the skin incisions were closed

SIEA flaps of female Sprague-Dawley rats were designed with 2.5 × 2.5-cm skin paddles and dissected as described previously [13] (Fig. 1, upper left panel). After the proximal and distal vasculature was controlled with vessel clamps, the flap pedicle was divided (Fig. 1, upper middle panel and upper right panel). During the ex vivo period, the afferent artery to the flap was catheterized with a 0.4 mm micro cannula (Kent Scientific, Torrington, CT, USA), while leaving the efferent artery unclamped and flushed with warm phosphate-buffered saline (PBS) to allow drainage of blood from the flap capillary system (Fig. 1, lower left panel). Subsequently, recombinant virus or PBS was perfused through the afferent artery with the efferent artery clamped. The virus was allowed to incubate within the ex vivo flap for 1 h at 37 °C. Afterwards, the efferent artery clamp was released and the flap was flushed manually with warm PBS to remove any unincorporated virus (Fig. 1, lower middle panel). The native vessels were re-anastomosed using 10–0 nylon sutures. The flap was oriented in its original position in the groin and skin incisions were closed with 5–0 nylon sutures (Fig. 1, lower right panel). The rats were placed in isolated cages in a biosafety level 2 animal care room.

### CDglyTK fusion gene expression within the flap and flap-bed interface

SIEA flaps were infected with LV-CMV-CDglyTK (5*10^8plaque forming units [pfu], *n* = 3), LV-CMV-GFP (5*10^8 pfu, *n* = 3), or PBS (*n* = 3). Animals were euthanized 3 days after infection and the flap and underlying flap bed along with the flap pedicle were harvested. Total RNA and protein were extracted, and mRNA and protein expression of the CDglyTK gene were detected by RT-PCR and western blot analysis, respectively.

### Therapeutic effects of LV-CDglyTK transfection of SIEA flaps and systemic toxicity

SIEA flaps in three groups of Sprague-Dawley rats were raised and SHZ-88 cells (1 × 10^7^) were injected subcutaneously into the flap immediately before transfection. Flaps were treated with 300 μL of LV-CMV-CDglyTK (5*10^8 pfu, *n* = 6), LV-CMV-GFP (5*10^8 pfu, *n* = 6), or PBS (*n* = 6). Next, an intraperitoneal injection of 5-FC + GCV (500 mg/kg + 50 mg/kg, respectively) was administered daily from the first post-operative day until the 21st day.

To assess systemic toxicity, serum was collected every week and analyzed for alanine transaminase (ALT) and aspartate transaminase (AST). Once a tumor nodule was palpable, tumor dimensions were measured every fifth day and tumor volume was calculated using the following formula: tumor volume (mm^3^) = width^2^ × length × 0.5, and tumor growth curves were generated. Animals were euthanized on Day 42 and weighed. Tumors and internal organs were harvested, fixed in 10% formalin for 24 h, embedded in paraffin and sectioned at 5-μm, and hematoxylin and eosin (H&E) staining of tumor tissues was performed for histological examination. The apoptosis in vivo tumor tissues was measured using TUNEL assay kit (Beyotime, Beyotime Biotechnology, China) according to the manufacturer’s protocol. The percentage of apoptotic cells was then analyzed by Image-pro plus 6.0 (Media Cybernetics, Inc., Rockville, MD, USA). Systemic toxicities of recombinant lentivirus and prodrugs were also determined by examining histological changes through H&E staining in the heart, lung, liver, spleen, kidney, and small intestine of Sprague-Dawley rats following treatment.

To detect systemic viral biodistribution, rats were culled at 7 days after transfection, and internal organs (heart, lung, liver, spleen, kidney, and small intestine) were harvested, followed by RNA extraction and RT-PCR determination of double suicide gene expression.

### Statistical analysis

Experimental data are presented as the mean ± standard deviation (SD). Differences between each group were processed by one-way analysis of variance and Tukey’s test using GraphPad Prism v5.0 (GraphPad Software, San Diego, CA, USA). *P*-values less than 0.05 were considered statistically significant.

## Results

### Virus titer determination

Forty-eight hours after the three plasmids were transferred into HEK293 cells, we found that over 95% of cells exhibited green fluorescence, which indicated the recombinant lentivirus was successfully constructed. Regarding determination of virus titer, we found two cells infected with LV-CDglyTK and one cell transfected with LV-GFP expressed GFP using 10^− 5^ μL recombined lentivirus. We determined that the virus titers of LV-CDglyTK and LV-GFP were 2E+ 8 TU/mL and 1E+ 8 TU/mL, respectively.

### Lentivirus-mediated transduction efficiency and expression of CDglyTK gene in SHZ-88 cells

**Fig. 2:**
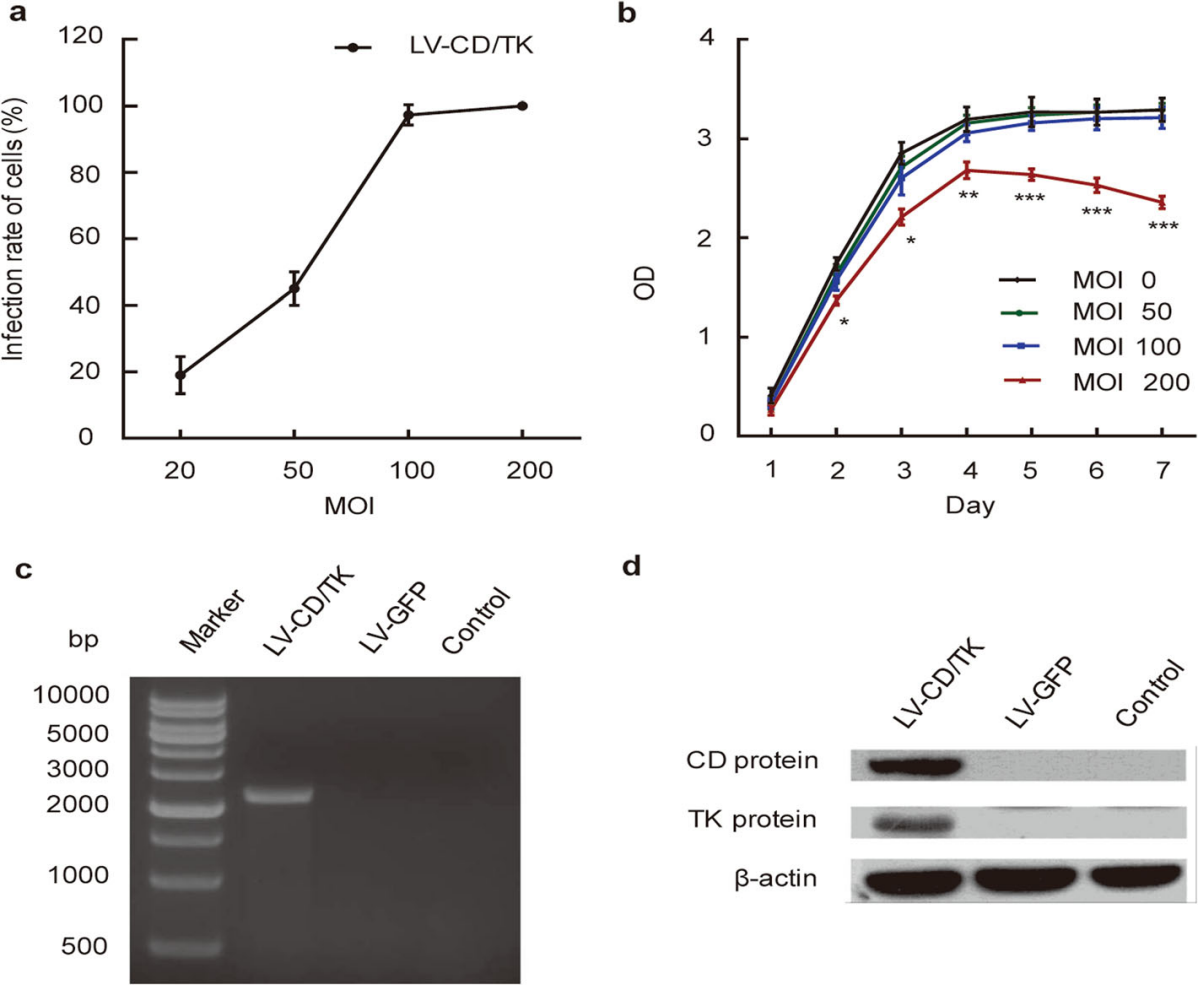
Transduction efficiency and expression of lentivirus-mediated CDglyTK gene in SHZ-88 cells. **a** Lentivirus-delivered gene transfer efficiencies in SHZ-88 cells. **b** The effect of the recombined lentivirus on growth of transfected cells. **c** Reverse transcriptase polymerase chain reaction analysis of CD/TK fusion gene expression in transfected cells. **d** Protein expression of the CD/TK double suicide gene by western blot in transfected cells. Data are presented as the mean ± SD, *n* = 3. *P < 0.05; **P < 0.01; ***P < 0.001. Three replicates were done in each experiment. LV-CD/TK, SHZ-88 cells transfected with lentivirus-mediated CDglyTK gene; LV-GFP, SHZ-88 cells transfected with empty lentivirus; Control, non-transfected SHZ-88 cells; CD, cytosine deaminase; TK, thymidine kinase

To examine lentivirus-mediated gene transfer efficiencies, cells from the SHZ-88 rat breast cancer cell line were infected with LV-CMV-CDglyTK and LV-CMV-GFP at various MOI, and GFP gene expression was observed by fluorescence microscopy. Using recombinant lentivirus at a MOI of 20, we found 19.67 ± 4.73% of cells expressed GFP. At MOIs of 100 and 200, we found 97.33 ± 3.06% and 99.33 ± 0.58% of cells exhibited green fluorescence, respectively (Fig. 2a).

Based on cell viability assay using CCK-8, we found that the growth of transfected cells was not affected when the MOI of recombined virus was less than 100 (*p* > 0.05); however, at MOIs over 200, cellular toxicity significantly inhibited growth of transfected cells (Fig. 2b).

Using RT-PCR analysis, we detected the expected 2400 base pair fragment of the CD/TK fusion gene in cells transfected with LV-CMV-CDglyTK, but not in cells of either the empty lentivirus-infected or control groups (Fig. 2c). We found using western blot analysis that predicted protein bands of approximately 48 and 25 kilodaltons were detected by anti-CD and anti-TK antibodies respectively, in cells of the target gene-infected group, but not in the two other groups (Fig. 2d).

### Effects of the double suicide CD/TK and prodrug system on killing efficacy on SHZ-88 cells

**Fig. 3:**
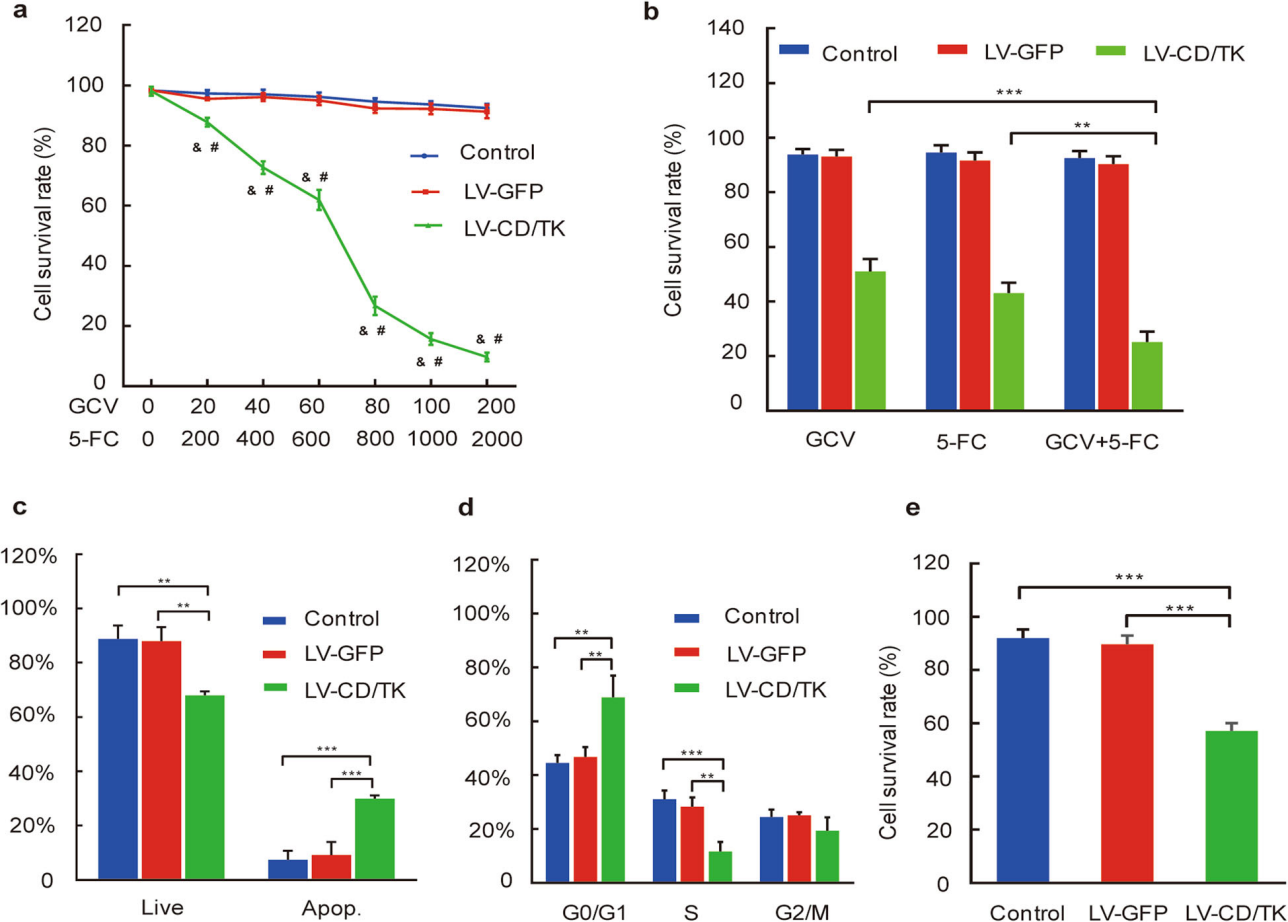
Effects of the double suicide CD/TK on the selective killing efficacy on SHZ-88 cells. **a** The cytotoxic effects of ganciclovir (GCV) and 5-flucytosine (5-FC) to transgenic cells. **b** The cytotoxic effects of different prodrugs to transgenic cells. **c** Cell apoptosis assay of 5-FC + GCV on transgenic cells. **d** Cell cycle analysis of 5-FC + GCV on transgenic cells. **e** Bystander effect analysis of the CD/TK fusion gene and prodrug system on SHZ-88 cells. Data are expressed as the mean ± SD, *n* = 3. & *P* < 0.001 compared to the LV-GFP group; #*P* < 0.001 compared to the control group; **P < 0.01; ***P < 0.001; LV-CD/TK, CDglyTK fusion gene transfected group; LV-GFP, empty lentivirus transfected group; Control, the untransfected group. Three replicates were done in each experiment

To analyze the cytotoxic effects of 5-FC and GCV to CDglyTK-expressing cells, SHZ-88 cells were transfected with LV-CDglyTK at an MOI of 100 and were then exposed to various concentrations of 5-FC and/or GCV for 48 h. As shown in Fig. 3a, compared to that found in the negative control group and the blank control group, the cell survival rate of the LV-CDglyTK group was markedly decreased following exposure to the prodrugs 5-FC and GCV (all *P* < 0.05), and this observed decrease in cell survival rate was dose-dependent. Administration of 800 mg/L 5-FC plus 80 mg/L GCV, the survival rate of SHZ-88 cells infected with LV-CDglyTK was significantly reduced (26.74 ± 3.05%), while the survival rates of the two control groups (negative and blank) showed little change (94.59 ± 1.08% and 92.33 ± 1.47%, respectively).

To understand the effect of the two prodrugs alone or in combination on the growth of SHZ-88 cells, we cultured CDglyTK-expressing cells in the presence of 5-FC (800 mg/L) or GCV (80 mg/L) alone or in combination for 48 h, and found that the survival rate of cells treated with a combination of 5-FC and GCV was strongly decreased (25.18 ± 3.80%) compared to that found in the 5-FC group (43.01 ± 3.87%) and the GCV group (51.03 ± 4.59%) (*P* < 0.01; Fig. 3b). This finding indicates that the inhibitory effect from the combined use of these two prodrugs on SHZ-88 cells infected with LV-CDglyTK was much stronger compared to either drug used alone.

Correspondingly, the percentage of apoptotic cells in the LV-CDglyTK group was increased (29.92 ± 1.16%) compared to that in the negative control group (9.20 ± 4.71%) and the blank control group (7.43 ± 3.35%). These results show that viability of SHZ-88 cells infected with LV-CDglyTK was significantly inhibited when treated with the prodrugs 5-FC and GCV at respective concentrations of 600 mg/L and 60 mg/L (*P* < 0.01; Fig. 3c).

Based on our findings using a cell cycle assay, we found that the percentage of cells of the LV-CDglyTK group was significantly increased in the G1 phase (68.97 ± 7.92%) and decreased in the S phase (11.73 ± 3.35%) compared to those found in the negative control group (46.77 ± 3.67% and 28.23 ± 3.45%, respectively) and the blank control group (44.50 ± 2.88% and 31.07 ± 3.20%, respectively) (*P* < 0.01; Fig. 3d). These results reveal that the cell cycle was arrested at the G1 phase following 48 h exposure to 5-FC (600 ml/L) and GCV (60 mg/L) in SHZ-88 cells transfected with LV-CDglyTK.

Based on our findings using CCK-8, the supernatant from the experimental group, which was treated with GCV and 5-FC, decreased growth of normal SHZ-88 cells (54.43 ± 3.00%), but the supernatant from the negative control group (86.56 ± 3.26%) and blank control group (88.74 ± 3.29%) did not have an obvious effect on normal cell growth, a finding which indicates the double suicide CD/TK gene and prodrug system showed a significant bystander effect on SHZ-88 cells (*P* < 0.001; Fig. 3e).

### Expression of the CD/TK double suicide gene in gene-modified flaps

**Fig. 4:**
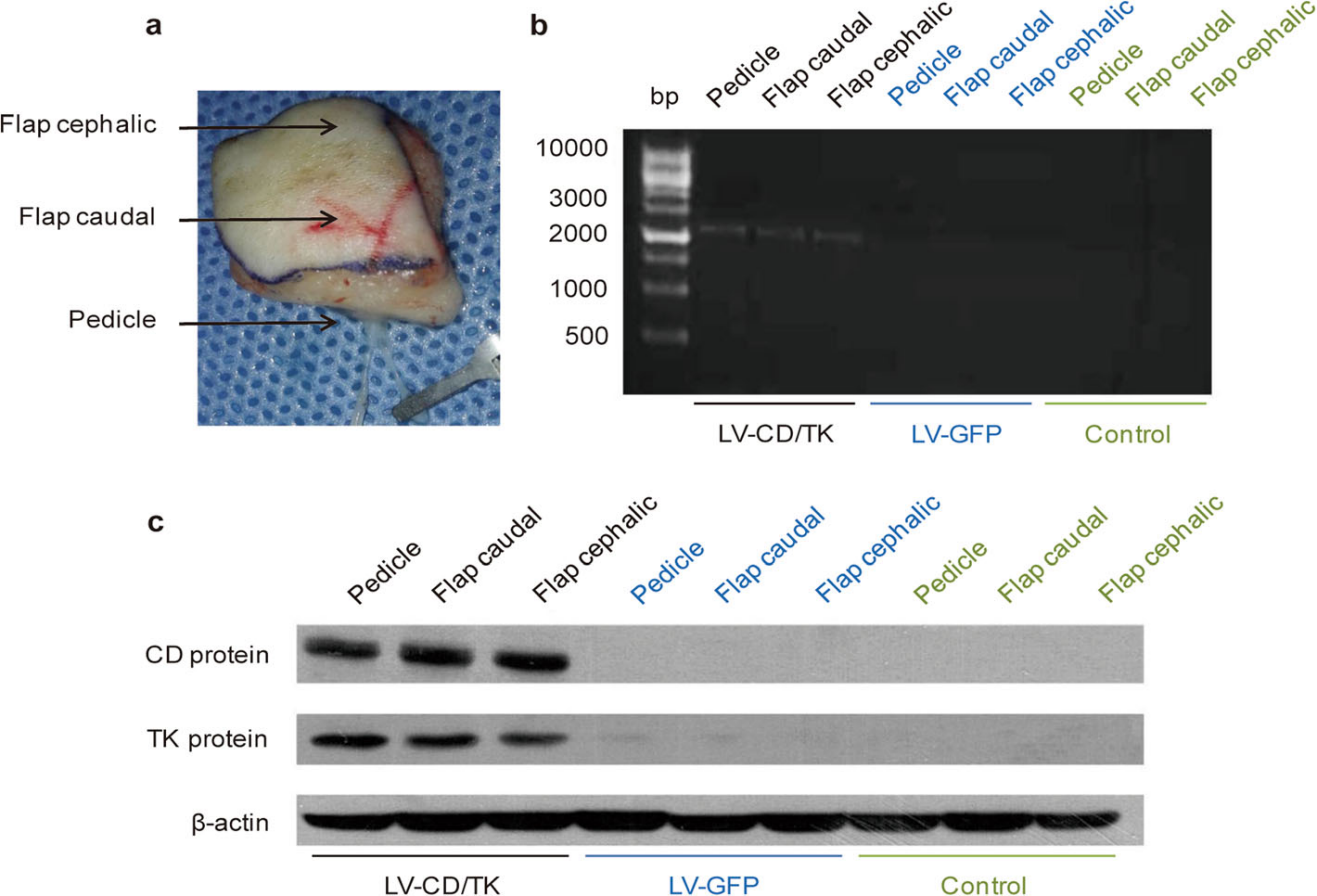
Expression of a CDglyTK fusion gene in gene-modified superficial inferior epigastric (SIEA) flaps. **a** Position of the pedicle, flap caudal, and flap cephalic in the free SIEA flap. **b** Reverse transcriptase polymerase chain reaction analysis of CD/TK fusion gene in SIEA flaps transfected with different recombinant lentivirus. **c** Protein expression of CD/TK double suicide gene by western blot in transfected flaps. Data was shown as the mean ± SD of three independent samples. LV-CDglyTK, flaps transfected with lentivirus-mediated CDglyTK gene; LV-GFP, flaps transfected with empty lentivirus; Control, non-transfected flaps

At 7 days post-operation, we found using RT-PCR and western blot assay that expression of the CD/TK fusion gene was detected in the SIEA flap including the cephalic flap, pedicle, and caudal flap tissues (Fig. 4a) of the experimental group, but was undetectable in flaps of the negative and blank control groups (Fig. 4b, c). In addition, no evident expression of the CD/TK gene was detected in the underlying flap bed in the experimental group (Fig. 4b, c).

### Lentivirus-mediated double suicide CDglyTK fusion gene by intra-arterial perfusion and prodrug system effectively suppressed rat transplanted breast cancer in vivo

**Fig. 5:**
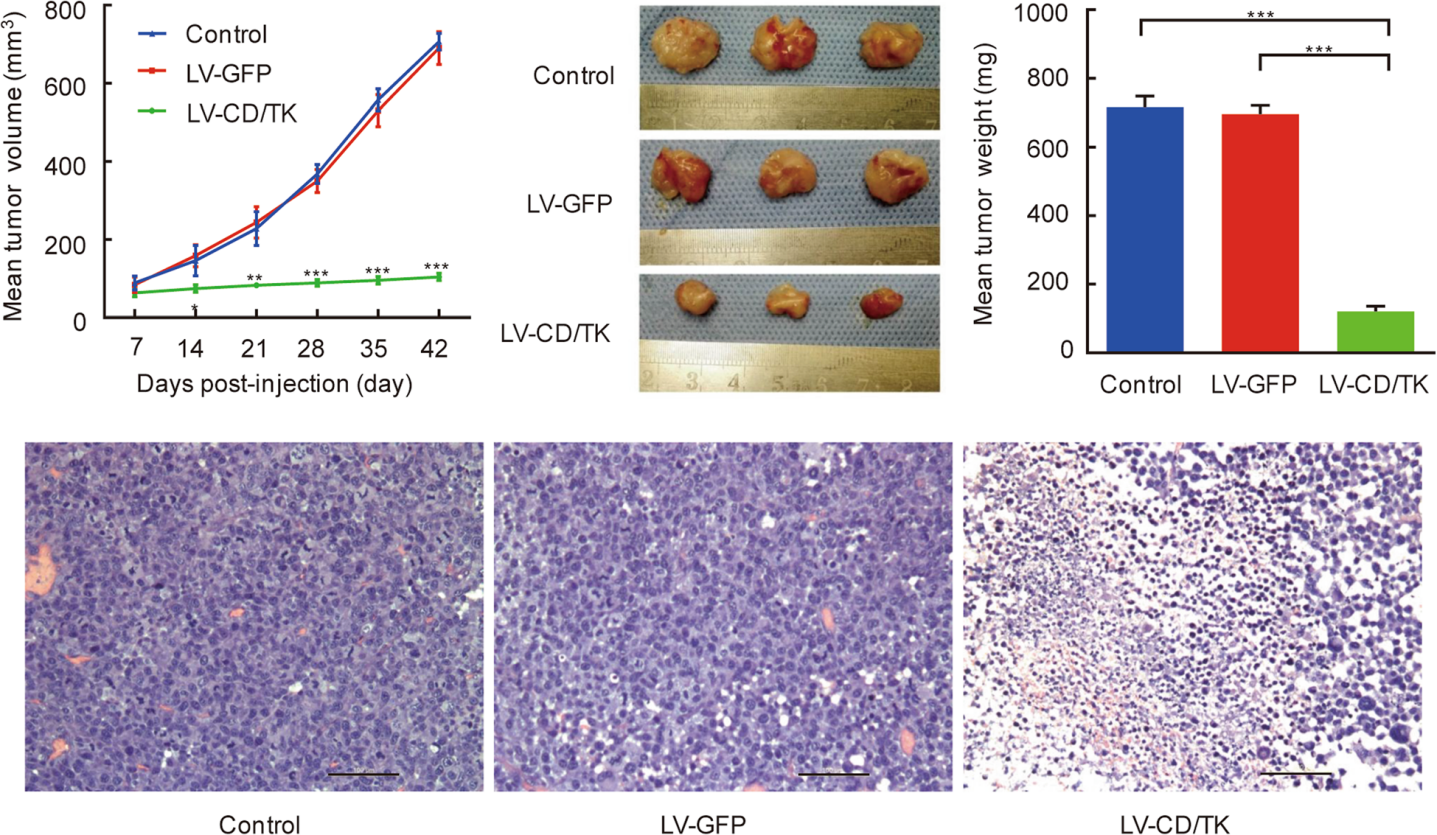
The lentivirus-mediated CDglyTK fusion gene by intra-artery perfusion effectively suppresses transplanted breast cancer in vivo. (upper left panel) Tumor growth curve. (upper middle panel) Appearance and (upper right panel) weight of transplanted tumors at 42 days after treatment. (lower panel) Histology analysis of the transplanted tumors by hematoxylin and eosin (H&E) staining. Scale bar: 100 μm. Data was shown as the mean ± SD of six independent samples. **P < 0.01; ***P < 0.001. LV-CDglyTK, flaps transfected with lentivirus-mediated CDglyTK gene; LV-GFP, flaps transfected with empty lentivirus; Control, non-transfected flaps

**Fig. 6:**
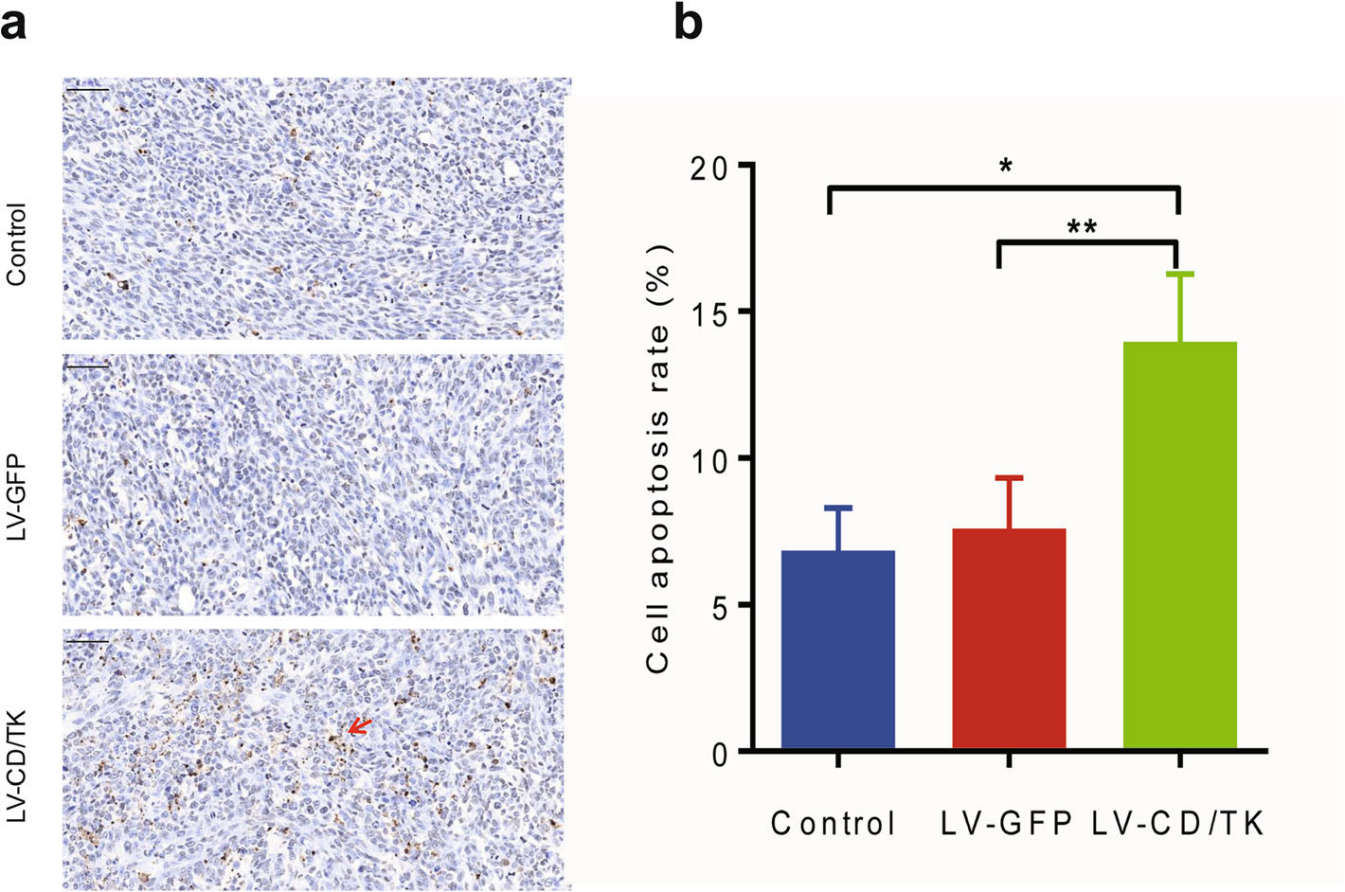
The apoptosis assay in vivo tumor tissues caused by active drug converted from prodrug using TUNEL assay kit. **a** Cell apoptosis assay of the transplanted tumors by TUNEL assay. Red arrowhead indicated the positive apoptosis cells in the tumor tissue. **b** The percentage of apoptotic cells in tumor tissue was analyzed by Image-pro plus 6.0. Data was shown as the mean ± SD of six independent samples. Scale bar: 50 μm. *P < 0.05; **P < 0.01. LV-CD/TK, flaps transfected with lentivirus-mediated CDglyTK gene; LV-GFP, flaps transfected with empty lentivirus; Control, non-transfected flaps

Following engraftment of rat breast cancer cells in transfected flaps and prodrug treatment, we found that tumor volume growth was significantly reduced in the experimental group compared to that of the two control groups (Fig. 5, upper left panel). At 42 days after treatment, the transplanted tumor volume (mm^3^) and tumor weight (mg) in the experimental group (104.00 ± 7.53 and 122.25 ± 6.93, respectively) were predictably lower than those in the negative control group (690.10 ± 41.95 and 693.56 ± 26.91, respectively) and blank control group (706.57 ± 21.22 and 720.17 ± 23.15, respectively) (*P* < 0.01; Fig. 5, upper middle and upper right panels). In addition, we found that the tumor inhibition rate in the experimental group (83.02%) was significantly higher compared to those found in the negative control groups (3.70%) (*P* < 0.01). The percentage of apoptotic cells in vivo tumor tissues in the LV-CDglyTK group was increased (13.98 ± 2.29%) compared to that in the negative control group (7.59 ± 1.73%) and the blank control group (6.84 ± 1.45%) (*P* < 0.05, Fig. 6). These results indicate that the cell apoptosis rate was increased significantly in vivo tumor tissues caused by active drug converted from prodrug. Further, H&E stained sections showed obvious necrosis in the transplanted tumor of the experimental group, while no abnormal histology was observed in either of the two control groups (Fig. 5, lower panel).

At 15 days and 42 days after SIEA flap transfection, the protein expression of CD/TK fusion gene was undetectable using immunohistochemical staining and western blot assay (Additional file 1: Figure S1 and Additional file 3:File S1). The result indicated that the free flap with lentivirus-mediated CDglyTK gene could play inhibition effects in vivo tumors via the bystander effect.

### Effects of lentivirus-mediated double suicide CDglyTK fusion gene by intra-arterial perfusion and prodrug system on systemic toxicity in rat breast cancer model

**Fig. 7:**
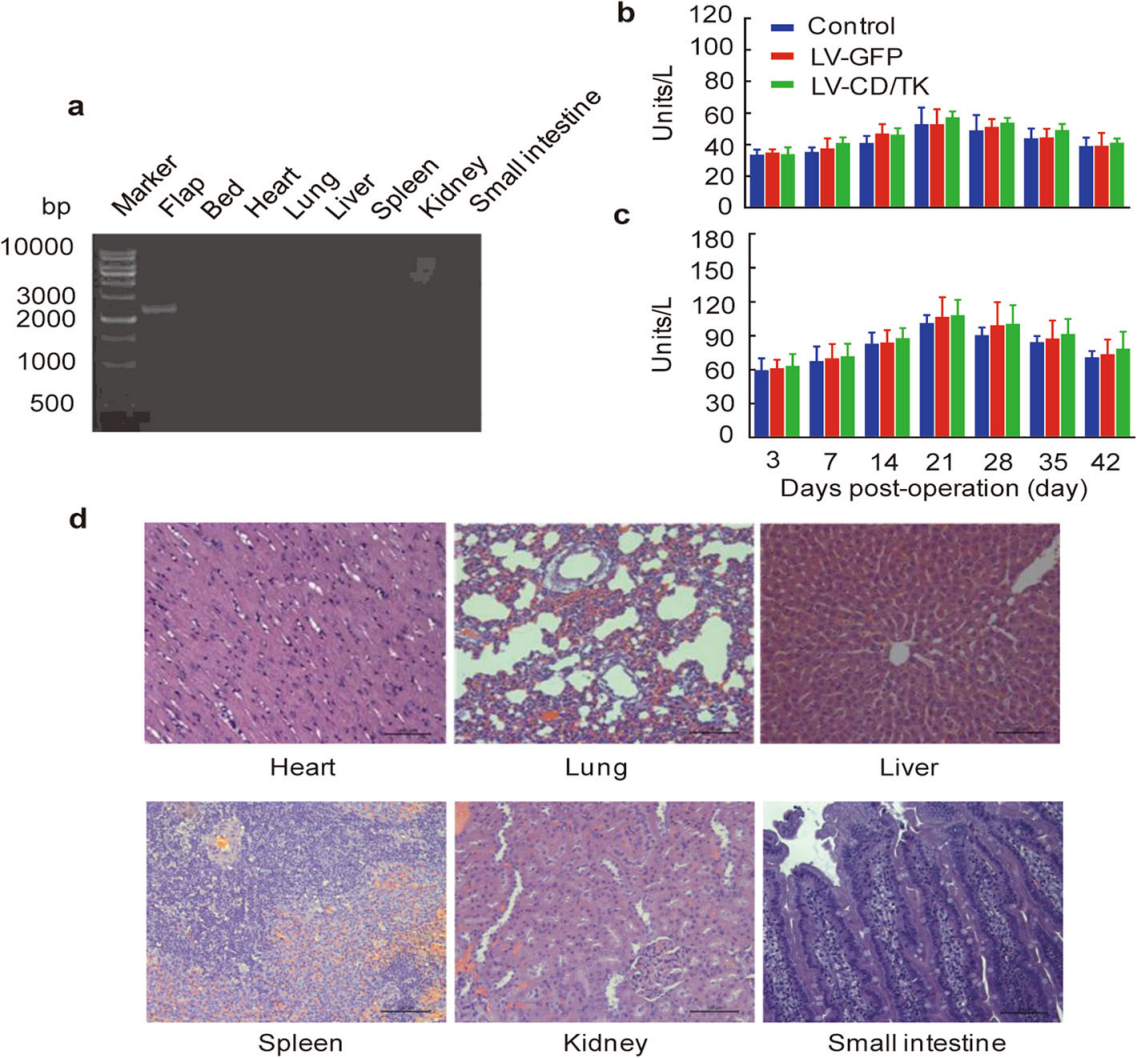
The lentivirus-mediated CDglyTK gene by intra-artery perfusion causes no discernable systemic toxic effects in vivo. **a** Reverse transcriptase polymerase chain reaction analysis of CD/TK fusion gene expression in the underlying flap bed and major internal organs. Analysis of serum **b** ALT and **c** AST over a 42-day post-operative period. **d** Assessment of the inflammatory response in major internal organs in the experimental group by hematoxylin and eosin (H&E) staining. Scale bar: 100 μm. Data was shown as the mean ± SD of six independent samples. LV-CDglyTK, flaps transfected with lentivirus-mediated CDglyTK gene; LV-GFP, flaps transfected with empty lentivirus; Control, non-transfected flaps

At 7 days after SIEA flap transfection, consistent expression of the CD/TK double suicide gene was detected in flap tissues by RT-PCR, but there was no evident expression identified in the underlying flap bed and major internal organs, including the heart, lung, liver, spleen, kidney, and small intestine (Fig. 7a). Over a 42 day post-operative period, we found no major fluctuations in serum levels of ALT and AST of the three groups (*P* > 0.05; Fig. 7b, c). In addition, we found no evidence of infiltration of inflammatory cells or metastasis of rat breast cancer cells in major internal organs by H&E staining (Fig. 7d). There was no significant difference in body weights of the animals among three groups (P > 0.05, Additional file 2 Figure S2 and Additional file 3: File S1).

## Discussion

Recently, gene therapy has proven to be an emerging and efficient means of cancer treatment [14, 15] and has entered clinical trials. A phase III clinical trial of suicide gene therapy using retrovirus-mediated transduction in glioblastoma failed to show improvements in survival outcomes by systemic viral administration owing to a failure to achieve a therapeutic dose of transgene expression at the site of disease [8, 16]. Free flaps may provide an alternative approach to systemic viral administration. In addition to their reconstructive function, free flaps can endure a relatively longer ischemic time (from 35 min to more than 1 h), providing a greater therapeutic probability for modifying the flap with the desired gene by intravascular perfusion [17–19]. Kobayashi et al. found that intravascular perfusion of recombinant lentivirus into free flaps was more efficient and localized compared to intramuscular and systemic delivery methods [20]. In addition, it was reported that an adequate balance of transgene expression and ischemic damage to flap tissue was achieved using a lentiviral incubation duration of 1 h at 37 °C under physiological pressure [21]. Moreover, if transfected flaps caused an adverse reaction at recipient sites, they can be easily removed [6]. Therefore, there is a promising future for free flap-mediated gene therapy in cancer treatment. Many gene therapy strategies in conjunction with free flaps have been recommended for use. A free flap as a vehicle for delivering a suicide gene is a promising therapeutic option for reducing the risk of local tumor recurrence; however, the efficacy and systemic toxicity of VDEPT delivered through free flaps required further investigation. In this study, we performed a preclinical evaluation of lentivirus-mediated CDglyTK gene and prodrug therapy in transplanted breast tumor by modifying SIEA flaps.

Gene delivery systems can affect both the intensity and duration of target gene expression either in infected cells or within the tissue of the free flap. Su et al. [12] reported that MCF7 human breast cancer cells transfected with an adenovirus-mediated CDglyTK double suicide gene with the cytomegalovirus promoter effectively expressed therapeutic transgenic products, which significantly suppressed proliferation of infected cells by pre-treatment with the prodrugs 5-FC and GCV. Further, the inhibitory effect of the adenovirus-mediated CDglyTK fusion double suicide gene on growth of human breast tumors continued for 18 days after the recombinant adenovirus was injected into the tumors in nude mice [12]. Compared to other vectors, lentivectors have the potential for a greater infection efficiency and more stable host genome integration of the target gene, leading to longer therapeutic gene expression. In fact, a transferred gene was continuously expressed in mesenchymal stem cells for 2 months after injection of lentivirus-mediated enhanced GFP into the cavity of mouse femoral bone in vivo [22, 23]. The tumor inhibition effect of neural stem cells transfected with a CD/TK fusion gene using lentivectors lasted 21 days in a glioma mouse model [24], and furthermore, lentivectors have been successfully used in clinical trials for the correction of adrenoleukodystrophy, β-thalassemia, and leukemia [25]. However, until our study, the transfection of a CDglyTK double suicide gene in any type of breast cancer cells with lentivectors has not been investigated.

In our study, we successfully produced a recombinant lentivirus containing the CDglyTK gene by a three-plasmid lentivirus packaging system, and not only found that the CDglyTK fusion gene was effectively expressed in rat SHZ-88 breast cancer cells infected with LV-CMV-CDglyTK, but also demonstrated a decreased cell survival rate in SHZ-88 cells when treated with 5-FC and GCV in a dose-dependent manner in vitro. Furthermore, the killing efficiency of transfected SHZ-88 cells from the combined use of 5-FC and GCV was much higher than that found using either drug alone, which indicates the inhibitory effect of the CDglyTK fusion gene combined with prodrug co-treatment was significantly enhanced. Our results also indicate that the double suicide gene system exerted an inhibitory effect on CDglyTK-transfected SHZ-88 cells by inducing apoptosis and G1/S cell cycle arrest. Previous research has shown that HSV-1 TK/GCV exhibited the largest therapeutic index, while CD/5-FC exhibited a stronger bystander effect [24]. In our study, we also found that the CDglyTK fusion gene and prodrug system has significant bystander cytotoxicity on SHZ-88 cells.

Genetically modified free flaps provide surgeons the opportunity to apply methods of adjuvant therapies to treat residual disease after radial resection. Seth et al. investigated the effects of an adenoviral-mediated TK gene and GCV therapy system in vivo using models of residual disease of rat glioma in a rat SIEA flap model, and determined that the transferred TK gene was expressed within the flap for up to 21 days [13]. In addition, tumor growth was inhibited and the median survival of the animal increased from 21 to 28 days. Recently, it was reported in rat models that the mean survival time of SHZ-88 cell subcutaneous transplantation was 39.7 ± 1.2 days [26]. In the current study, we observed an anti-tumor effect of the CD/TK double suicide gene and prodrug system on a rat transplanted breast cancer model for up to 42 days. Our results also demonstrated that the inhibition effect of a CD/TK double suicide gene in vivo by inducing apoptosis of tumor tissues could last until the end of the observation period, playing a therapeutic role by preventing tumor recurrence in the local area of transplanted flap. Further, no metastatic tumor was found in the main organs of the rats, demonstrating that the double suicide gene system prevents metastasis of rat transplanted breast cancer in vivo.

In addition to the marked inhibition of transplanted SHZ-88 breast cancer cells in the free SIEA flap, our study showed that CD/TK fusion gene expression was limited to the free SIEA flap tissue, and no viral sequence was detected in either the flap-bed interface or major internal organs following transfection of the SIEA flaps with LV-CDglyTK by intra-arterial perfusion. Furthermore, serum levels of the liver enzymes ALT and AST in rats remained normal and inflammatory infiltration of the major internal organs was not observed, findings which indicate the recombinant lentivirus and prodrugs did not cause any obvious systemic toxic effects to the rats.

## Conclusions

We demonstrated that regional intra-artery perfusion of free flaps using a lentivirus-mediated CDglyTK gene showed an effective long-lasting targeted therapeutic efficacy on transplanted breast cancer in rats without the general toxicity. Although further research is warranted before progressing to clinical trials, suicide gene transfection and drug treatment may be a promising future treatment regimen for breast cancer.

## Additional files

Additional file 1: **Figure S1**. Expression of a CDglyTK fusion gene in the tumor tissues at 15 days and at 42 days after SIEA flap transfection. (DOCX 6143 kb)

Additional file 2: **Figure S2**. The body weights of the animals of three groups over a 42 day post-operative period. (DOCX 1080 kb)

Additional file 3: **File S1** Methods and Figure legends of Supplementary Figures. (DOCX 15 kb)

## Data Availability

The datasets used and analysed during the current study are available from the corresponding author on reasonable request.

## Abbreviations

5-FC: 5-flucytosine
ALT: Alanine transaminase
AST: Aspartate transaminase
CCK-8: Cell Counting Kit-8
CD: Cytosine deaminase
CDglyTK: Cytosine deaminase-thymidine kinase
GCV: Ganciclovir
GFP: Green fluorescence protein
H&E: Hematoxylin and eosin
HSV-TK: Herpes simplex virus -thymidine kinase
MOI: Multiplicities of infection
PBS: Phosphate-buffered saline
pfu: Plaque forming units
RT-PCR: Reverse transcriptase polymerase chain reaction
SD: Mean ± standard deviation
SIEA: superficial inferior epigastric artery
VDEPT: Virus-directed enzyme prodrug therapy
