# Supplementary material for: Lentivirus-mediated CDglyTK gene-modified free flaps by intra-artery perfusion show targeted therapeutic efficacy in rat model of breast cancer
Source: BMC Cancer. 2019 Sep 14;19:921. doi: 10.1186/s12885-019-6111-5 (PMC6744674; doi:10.1186/s12885-019-6111-5)

**Additional file 1 Figure S1** Expression of a CDglyTK fusion gene in the tumor tissues at 15 days and at 42 days after SIEA flap transfection. (a) Immunohistochemical staining analysis of TK gene expression in the tumor tissues. (b) Protein expression of CD/TK double suicide gene by western blot in the tumor tissues. LV-CD/TK, flaps transfected with lentivirus-mediated CDglyTK gene; LV-GFP, flaps transfected with empty lentivirus; Control, non-transfected flaps.


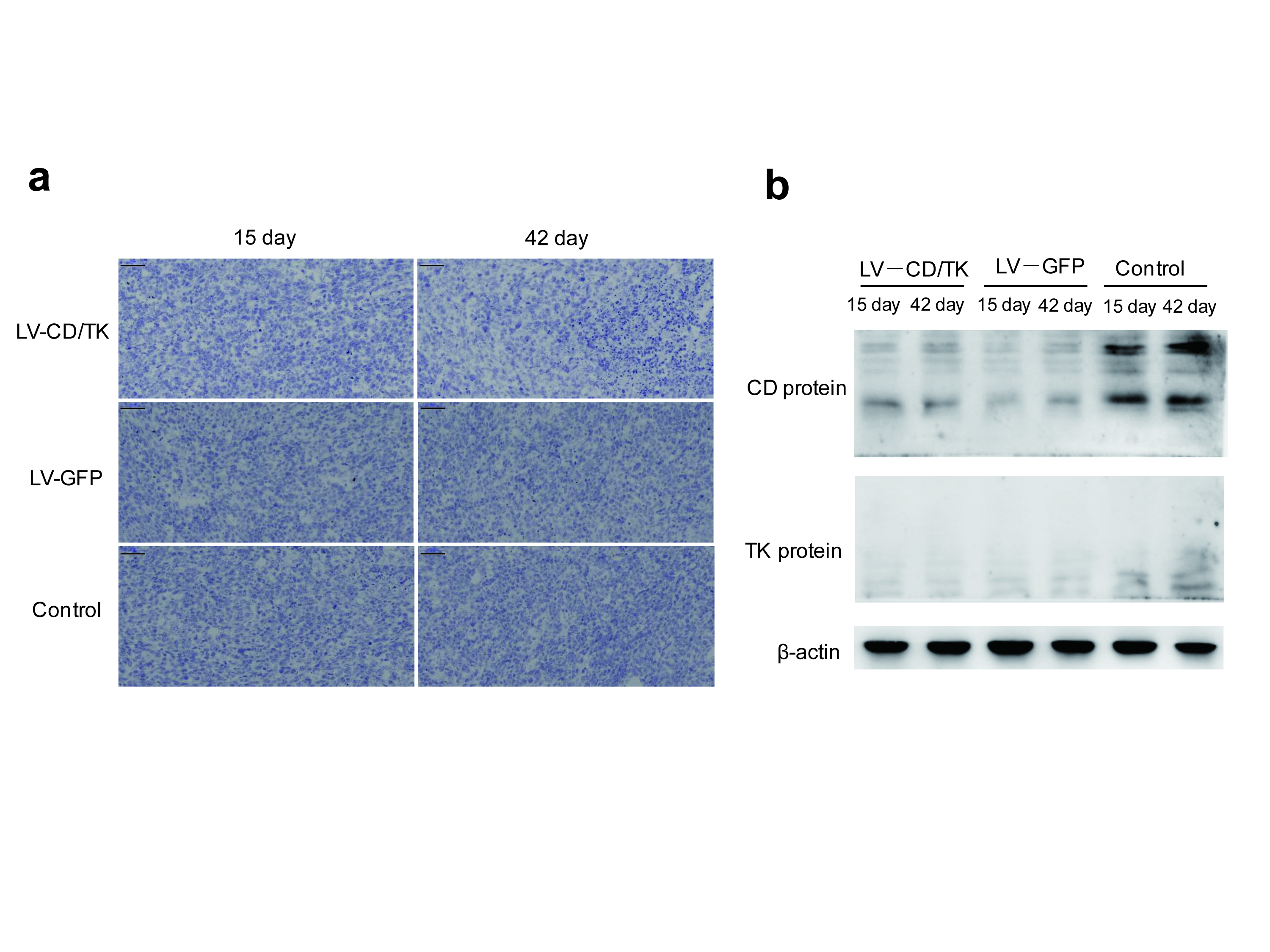

Supplement: Supplementary file 1 — Figure S1. Expression of a CDglyTK fusion gene in the tumor tissues at 15 days and at 42 days after SIEA flap transfection. (DOCX 6143 kb) [file 12885_2019_6111_MOESM1_ESM.docx]
