# Supplementary material for: Lentivirus-mediated CDglyTK gene-modified free flaps by intra-artery perfusion show targeted therapeutic efficacy in rat model of breast cancer
Source: BMC Cancer. 2019 Sep 14;19:921. doi: 10.1186/s12885-019-6111-5 (PMC6744674; doi:10.1186/s12885-019-6111-5)

**Additional file 2: Figure S2** The body weights of the animals of three groups over a 42 day post-operative period. Animals were euthanized on Day 42, and the bodies of these animals were weighed. Data was shown as the mean ± SD of six independent samples.LV-CD/TK, flaps transfected with lentivirus-mediated CDglyTK gene; LV-GFP, flaps transfected with empty lentivirus; Control, non-transfected flaps.


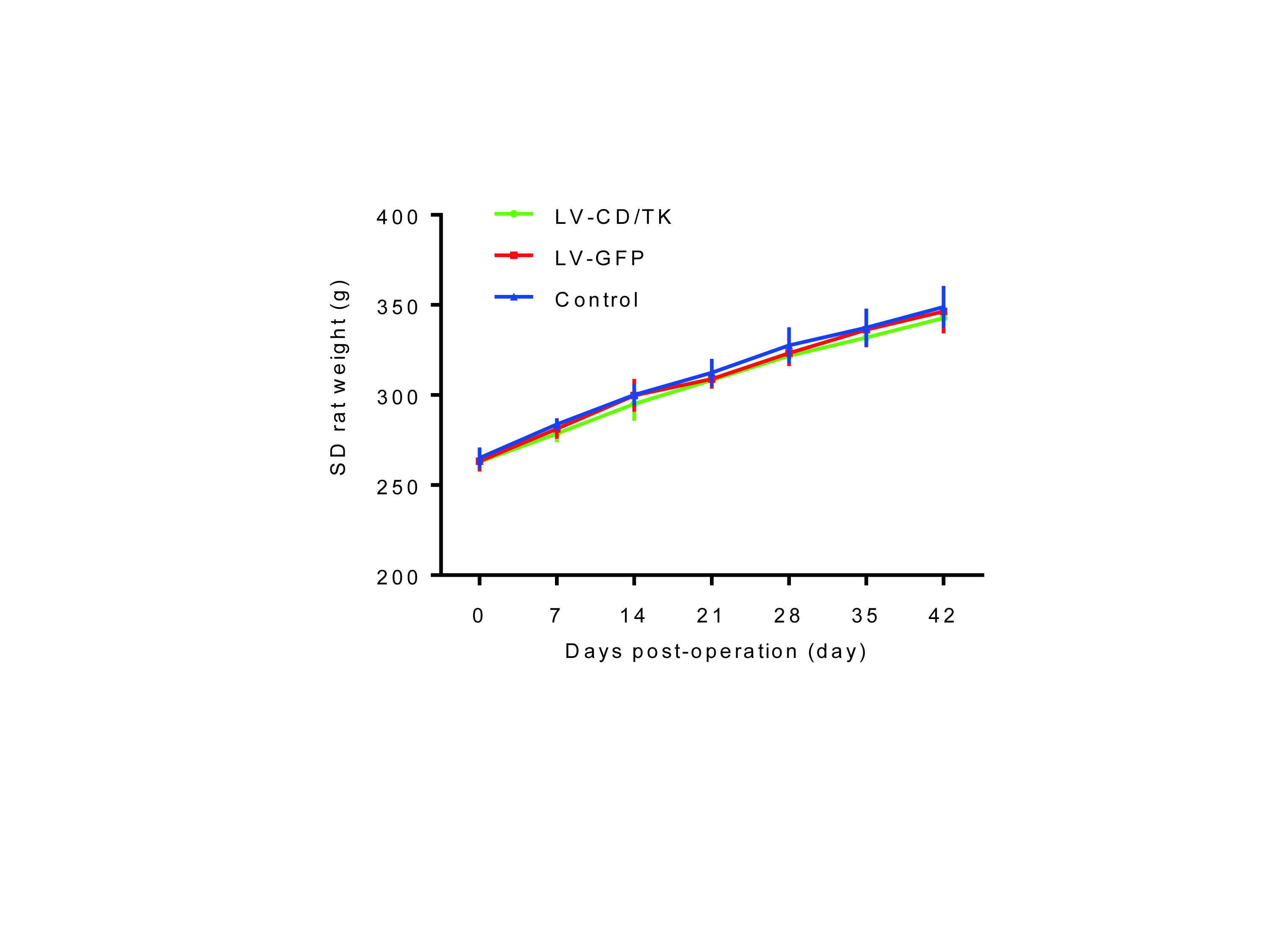

Supplement: Supplementary file 2 — Figure S2. The body weights of the animals of three groups over a 42 day post-operative period. (DOCX 1080 kb) [file 12885_2019_6111_MOESM2_ESM.docx]
