# Supplementary material for: Lentivirus-mediated CDglyTK gene-modified free flaps by intra-artery perfusion show targeted therapeutic efficacy in rat model of breast cancer
Source: BMC Cancer. 2019 Sep 14;19:921. doi: 10.1186/s12885-019-6111-5 (PMC6744674; doi:10.1186/s12885-019-6111-5)
Supplement: Supplementary file 3 — File S1 Methods and Figure legends of Supplementary Figures. (DOCX 15 kb) [file 12885_2019_6111_MOESM3_ESM.docx]

**Additional file 3: File S1** Methods and Figure legends of Supplementary Figures

**Methods**

**IHC and Western blot**

Animals were euthanized at 15 days and at 42 days after SIEA flap transfection, when the dimension of the tumors reached about 3 mm and at the end of the study respectively, tumor tissues were harvested. The samples of tumor tissues were fixed in 10% formalin, embedded in paraffin, sectioned at 5-µm, and the total protein was extracted. Immunohistochemical staining and western blot analysis were performed to detect the protein expression of the CDglyTK gene respectively.

Immunohistochemical studies were performed using standard procedures. TK gene was detected using specific TK polyclonal primary antibody (1:200, Santa Cruz Biotechnology, Santa Cruz, CA, USA). Secondary antibodies from the Two-Step Polymer Non-Biotin HRP Detection System for DAB (Golden Bridge International, Mukilteo, WA, USA) were used. Stained immunohistochemical sections were imaged with a Leica DM3000 microscope (Leica Microsystems Gmbh, Wetzlar, Germany).

Total proteins (15 μg/well) were electrophoresed by 10% sodium dodecyl sulfate polyacrylamide gel electrophoresis and transferred to polyvinylidene fluoride membranes at 200 mA for 2 h. CD polyclonal antibody (GeneTex Biotechnology, Irvine, CA, USA) or TK polyclonal antibody (Santa Cruz Biotechnology, Santa Cruz, CA, USA) were applied at 1:1000 dilutions, and a goat anti-rabbit or goat anti-mouse horseradish peroxidase (1:10 000, Jackson ImmunoResearch, West Grove, PA, USA) was used as the secondary antibody. Blots were detected using an ECL detection kit and a Gel Image system ver.4.00 (Tanon, Shanghai, China).
